# Metabolic Heterogeneity of Cancer Cells: An Interplay between HIF-1, GLUTs, and AMPK

**DOI:** 10.3390/cancers12040862

**Published:** 2020-04-02

**Authors:** Nurbubu T. Moldogazieva, Innokenty M. Mokhosoev, Alexander A. Terentiev

**Affiliations:** 1 Laboratory of Bioinformatics, I.M. Sechenov First Moscow State Medical University (Sechenov University), 119991 Moscow, Russia; 2 Department of Biochemistry and Molecular Biology, N.I. Pirogov Russian National Research Medical University, 117997 Moscow, Russia; imokhosoev@mail.ru (I.M.M.); aaterent@inbox.ru (A.A.T.)

**Keywords:** cancer metabolism, OXPHOS, HIF-1, AMPK, GLUTs

## Abstract

It has been long recognized that cancer cells reprogram their metabolism under hypoxia conditions due to a shift from oxidative phosphorylation (OXPHOS) to glycolysis in order to meet elevated requirements in energy and nutrients for proliferation, migration, and survival. However, data accumulated over recent years has increasingly provided evidence that cancer cells can revert from glycolysis to OXPHOS and maintain both reprogrammed and oxidative metabolism, even in the same tumor. This phenomenon, denoted as cancer cell metabolic plasticity or hybrid metabolism, depends on a tumor micro-environment that is highly heterogeneous and influenced by an intensity of vasculature and blood flow, oxygen concentration, and nutrient and energy supply, and requires regulatory interplay between multiple oncogenes, transcription factors, growth factors, and reactive oxygen species (ROS), among others. Hypoxia-inducible factor-1 (HIF-1) and AMP-activated protein kinase (AMPK) represent key modulators of a switch between reprogrammed and oxidative metabolism. The present review focuses on cross-talks between HIF-1, glucose transporters (GLUTs), and AMPK with other regulatory proteins including oncogenes such as c-Myc, p53, and KRAS; growth factor-initiated protein kinase B (PKB)/Akt, phosphatidyl-3-kinase (PI3K), and mTOR signaling pathways; and tumor suppressors such as liver kinase B1 (LKB1) and TSC1 in controlling cancer cell metabolism. The multiple switches between metabolic pathways can underlie chemo-resistance to conventional anti-cancer therapy and should be taken into account in choosing molecular targets to discover novel anti-cancer drugs.

## 1. Introduction

Cancer cells often suffer from hypoxia, nutrient (glucose and amino acid), and energy deprivation resulting from insufficient vasculature and blood supply [1]. These stress conditions are key factors imposed on proliferating tumor cells to trigger their malignant transformation and to enable them to overcome or escape antitumor immune surveillance and to avoid cellular senescence and apoptosis [2,3,4]. This results in tumor progression and aggressiveness, genetic instability, development of chemo- and radio-resistance, and poor prognosis [5,6]. 

Under physiological conditions, oxidative phosphorylation (OXPHOS), that is, coupling of oxidation reactions with mitochondrial electron transportation chain (ETC), is the most efficient form of ATP production, generating a much larger amount of energy as compared to anaerobic glycolysis; however, under hypoxic conditions, glycolysis is the only process that provides cells with energy [7,8]. In the hypoxic microenvironment, cancer growth is maintained by metabolic and bioenergetic reprogramming that is characterized by adaptive switch from OXPHOS to glycolysis, followed by excessive glucose consumption and lactate production. This phenomenon was first discovered by German scientist Otto Warburg in 1927, and was denoted as the Warburg effect by Efraim Racker in 1972 [9,10,11].

Molecular mechanisms underlying cancer cell tolerance to prolonged hypoxia and nutrient/energy starvation are very complex and can work both at transcriptional and post-translational levels. Hypoxia-inducible factor-1 (HIF-1) is a master regulator of cellular oxygen sensing and adaptation to hypoxia and ubiquitous transcriptional activator, which regulates the expression of numerous genes at DNA and epigenetic (chromatin remodeling/histone modification) levels [12,13,14]. The modulation of gene expression by HIF-1 causes alterations in mitochondrial oxidative metabolism, glucose uptake and oxidation, energy production, and angiogenesis in order to enable cancer cell proliferation, migration, and survival.

However, a large body of data has shown that most tumors grow in their interaction with a highly heterogeneous microenvironment with different densities of blood and lymph vessels, amount and types of infiltrating cells, extracellular matrix composition, and content of signaling molecules, among others [15] Moreover, many tumors are not monoclonal despite originating from a single cell; instead, they are composed of multiple distinct clones that can be differentiated by morphological and phenotypic features, and can vary depending on cancer type, cancer stage, and treatment regimes, among other factors [16,17,18]. This phenomenon denoted as tumor heterogeneity implies that a heterogeneous population of various cell types with distinct gene expression and metabolic profiles, as well as proliferative, angiogenic, and metastatic potential, co-exist within a definite tumor.

Furthermore, results of experimental, bioinformatics and computer/mathematical modeling approaches increasingly evidence that cancer cells do not fully rely on glycolysis; instead, they preserve oxidative metabolism [19,20]. This indicates that cancer cells acquire hybrid or heterogeneous metabolism, which enables them to use both glycolysis and OXPHOS as sources of ATP and that oxidative catabolic pathways including tricarboxylic acid (TCA) cycle (Krebs cycle), oxidative decarboxylation of pyruvate, glutaminolysis, and fatty acid β-oxidation (FAO) can remain functional as sources of reducing equivalents (NADH and FADH_2_), as well as carbon and nitrogen [20]. Moreover, multiple switches between the metabolic pathways can exist depending on various intrinsic and extrinsic factors such as nutrient and energy availability; micro-environmental and dietary factors; and clinico-pathological characteristics such as tumor stage, histological type, differentiation grade, lymph node involvement, and depth of invasion, among others. Importantly, the metabolic switches impact tumor outcome and patient responsiveness to anti-cancer therapy.

To provide cancer cell metabolic plasticity, induction of numerous genes and activation or inhibition of multiple oncogenes, growth factors, and tumor suppressors are required [21]. A crucial role in this phenomenon belongs to interplay between HIF-1 and AMP-activated protein kinase (AMPK), an energy sensor and master regulator of cellular metabolism and bioenergetics. AMPK is a heterotrimeric serine/threonine kinase that is activated in response to decrease in AMP/ATP ratio in order to provide ATP production through both glycolysis and OXPHOS [22]. In general, AMPK maintains ATP level in cells due to a switch from anabolic to catabolic metabolism through the stimulation of glucose uptake, aerobic glycolysis, and mitochondrial oxidative metabolism, mainly due to β-oxidation of fatty acids [23]. 

Additionally, both hypoxia and nutrient deprivation can cause elevated generation of reactive oxygen species (ROS) by mitochondrial ETC and Nox family NADPH oxidases, resulting in oxidative stress and alterations in cell signaling pathways [24]. Variety of ROS types can affect the activities of both HIF-1 and AMPK along with intracellular effectors of cell signaling pathways and transcription factors to trigger cancer progression and metastasis under hypoxia, nutrient/energy starvation, and oxidative stress conditions [25].

This review focuses on the recent advancements in understanding mechanisms, which underlie the ability of cancer cells to maintain metabolic heterogeneity, both metabolic/bioenergetic reprogramming and oxidative metabolism, for proliferation, invasion, and metastasis. We demonstrate here the importance of consideration of cross-talks between HIF-1 and AMPK, their interplay with glucose transporters (GLUTs), and influence on the expression of enzymes involved in glucose and fatty acid metabolism during cancer initiation and progression. Furthermore, we show that growth factor-initiated phosphatidyl-3-kinase (PI3K), protein kinase B (PKB)/Akt, and mammalian target of rapamycin (mTOR) cell signaling pathways, along with oncogenes and transcription factors such as KRAS, c-Myc, and p53 interplay with HIF-1 and AMPK, as well as ROS generation, to enable cancer cell metabolic plasticity. 

## 2. Hypoxia-Inducible Factors

The master regulators of oxygen homeostasis, HIFs, are evolutionarily conserved transcription factors that are expressed in all eukaryotes in three isoforms: HIF-1α, HIF-2α, and HIF-3α (reviewed by [26,27]). HIF-1α undergoes heterodimerization with HIF-1β, both containing basic helix-loop-helix (bHLH) domains along with Per-aryl hydrocarbon nuclear translocation (ARNT)-Sim homology (PAS) domain [28]. Under normoxia conditions, HIF-1α is destabilized through continuous degradation by ubiquitin-proteasome system (UPS) [29]. However, under acute hypoxia conditions, HIF-1α dimerizes with HIF-1β and becomes stabilized in order to undergo translocation into the nucleus and to bind to hypoxia response elements (HREs) on DNA for regulation of gene expression. This leads to the over-expression of key regulatory enzymes of glycolysis and pentose phosphate pathway (PPP), as well as to down-regulation or mutations in genes encoding pyruvate decarboxylase complex (PDC) and TCA cycle enzymes or ETC enzymatic complex I, as observed in various cancer types (reviewed by [30,31,32]). 

Under normoxia conditions, two proline residues of HIFα subunits have been shown to undergo hydroxylation by prolyl-hydroxylase domain proteins (PHDs), which exist in three isoforms, from PHD1 to PHD3, and have distinct functions [33]. Under chronic hypoxia conditions, over-expression and hyper-activation of PHDs followed by HIFα desensitization to protect cells from necrosis has been observed [34].

Hydroxylation of proline residues and acetylation of a lysine residue enable recognition of HIF1α, HIF2α, and HIF3α by von Hippel–Lindau protein (pVHL) and the recruitment of E3 ubiquitin ligase for the HIFα polyubiquitination and UPS-mediated degradation [35]. Genetic loss in the pVHL tumor suppressor has been shown to cause HIF-1 stabilization and activation, even under normoxia conditions followed by tumor cell proliferation and survival [36,37]. Additionally, hydroxylation of asparagine residue by the factor inhibiting HIF (FIH) prevents binding of coactivator p300/CBP, followed by a decrease in HIFα transcriptional activity [38]. 

With the use of the tumor metabolism modeling approach, it has been shown that in the hypoxic microenvironment, both intracellular and environmental factors contribute to metabolic reprogramming of cancer cells, and that various growth factor-initiated cell signaling cascades and transcription factors can affect HIF-1 activity [39]. An interplay between HIF-1 and a variety of oncogenes such as Ras, c-Myc, p53, AMPK, along with PKB/Akt, PI3K, and mTOR signaling pathways has been observed to control mitochondrial ETC functioning and energy production to maintain cancer cell proliferation and survival [40,41,42,43,44,45,46,47]. 

For example, an interplay between HIF-1α and p53, two transcription factors regulated by both E3 ubiquitin ligase and murine double minute 2 (Mdm2), in response to hypoxia during carcinogenesis has been found [48]. The p53 activation by gamma-rays used in cancer treatment triggers Mdm2-mediated HIF-1α UPS-mediated degradation. This leads to decrease in the peroxisome proliferator-activated receptor gamma co-activator 1β (PGC-1β) inhibition and promotes mitochondrial biogenesis [49]. Additionally, oncogenic KRAS induces HIF-1α and HIF-2α genes, which leads to decreased OXPHOS and ATP production and increased mitochondrial ROS generation in colon cancer cells [50]. Moreover, KRAS can enhance ROS generation by NADPH oxidases, for example, in a Rac1-Nox4-dependent manner [51].

Under hypoxic conditions, HIF-1α expression can associate with the growth factor expression. Indeed, signal transduction pathways initiated by binding of epidermal growth factor (EGF) and platelet-derived growth factor (PDGF) to their membrane-bound receptors, receptor tyrosine kinases (RTKs), activate Ras/PI3K/phosphatase and tensin homolog (PTEN)/Akt and mTOR signaling to induce HIF-1 and c-Myc expression [52,53]. For example, PI3K/Akt/mTOR pathway-mediated stimulation of HIF-1 mRNA translation through the activation of two downstream targets of mTOR, p70SK6, and 4E-BP1 in breast cancer cells has been observed [37]. 

Additionally, Krüppel-like factor 5 (KLP5) promotes non-small cell lung cancer (NSCLC) cell apoptosis via direct suppression of HIF-1α and glycolysis. The over-expression of KLF5 promotes cancer cell survival and hypoxia-induced cisplatin resistance through the activation of the PI3K/Akt/mTOR pathway [54]. Further, mechanism of PDC inhibition and reduction in oxidative decarboxylation of pyruvate to acetyl-CoA in response to hypoxia may involve the mitochondrial PKB/Akt accumulation and pyruvate dehydrogenase kinase 1 (PDK1) phosphorylation at Thr-346. This triggers glycolysis to maintain tumor cell proliferation, antagonizing apoptosis and autophagy [55]. 

Moreover, in tumor patients, HIF-1α expression has been observed to correlate with vascular endothelial growth factor (VEGF) over-expression, along with tumor stage and vascularization, lymphatic invasion, and metastasis, as shown in many cancer types such as esophageal squamous cell cancer, as well as colorectal and hepatocellular carcinomas [56,57,58,59]. Moreover, the inhibition of HIF-1α and VEGF expression both at mRNA and protein levels has been reported to suppress tumor growth, whereas targeting mTOR/HIF-1α/VEGF can be considered as a promising strategy in anti-cancer therapy [58]. For example, the inhibition of human cervical cancer growth and the enhancement of tumor radio-sensitivity can be achieved by down-regulation of HIF-1α and VEGF and up-regulation of p53 [60]. In an animal model of cervical cancer, the tumor growth inhibition by formononetin and cisplatin has been associated with decrease in HIF-1α and VEGF expression [61]. Targeting HIF-2 in clear cell renal carcinoma (ccRCC) led to dissociation of HIF-2α/HIF-1β dimer to suppress tumor growth [62]. 

Furthermore, metabolomics and quantitative proteomics approaches have shown that the mitochondrial NAD^+^-dependent deacetylase family of enzymes, sirtuins (SIRTs), can alter cellular metabolism and revert the Warburg effect in tumor cells [63]. For example, SIRT3 over-expression has been implicated in kidney cancer growth inhibition and maintaining mitochondrial homeostasis, as well as modulating ROS production to sensitize biomolecules and cells to oxidative damage [64], whereas low SIRT3 level is associated with poor differentiation and unfavorable prognosis in primary hepatocellular carcinoma (HCC) [65]. Moreover, anti-cancer agent etoposide-induced genotoxic-mediated cancer cell apoptosis has been shown to correlate with both SIRT1 and SIRT3 over-expression [66].

SIRTs can regulate mitochondrial metabolism in response to diverse nutrient signals through the deacetylation of various proteins including TCA cycle enzyme isocitrate dehydrogenase-2 (IDH2), ETC enzymatic complexes, and antioxidant enzyme Mn-superoxide dismutase (SOD2). Thus, they promote TCA cycle and ETC functioning, and reduce ROS generation and oxidative stress, while a loss of SIRTs enhances metabolic reprogramming in cancer cells through destabilization of HIF-1α to down-regulate glycolytic genes [67]. For example, in breast cancer, the over-expression of SIRT3 decreases the rate of glycolysis and inhibited cell proliferation followed by tumor suppression, whereas down-regulation of SIRT3 increased ROS production leading to HIF-1α stabilization and upregulation of HIF-1α target genes [68]. Seemingly, SIRT3 can regulate metabolic reprogramming in cancer cells also through deacetylation of p53 transcription factor at Lys-320 and Lys-382 residues to promote its UPS-mediated degradation, as shown in phosphatase and tensin homolog (PTEN)-deficient non-small cell lung cancer cells [69]. 

## 3. Interplay between HIF-1 and Facilitative Glucose Transporters 

The phenotypic hallmark of more than 90% of primary and metastatic tumors is an increase in glucose uptake from the blood, which occurs to compensate a low ATP yield via glycolysis and to a great extent depends on facilitative glucose transporters (GLUTs) encoded by the *SLC2A* gene family [70]. This family comprises 14 members, GLUT1–14, grouped into four classes on the basis of sequence similarity. Additionally, GLUTs vary in their affinity to glucose, regulation, tissue distribution, and expression level under both physiological and pathological conditions. 

Under physiological conditions, GLUT4 is a major insulin-sensitive glucose transporter. TBC1D1, Tre2/Bub2/Cdc15 (TBC) domain family member 1 protein, can regulate insulin-stimulated GLUT4 translocation into a mammalian cell membrane, thereby triggering glucose uptake [71]. TBC1D1 is a Rab-GTPase-activating protein and contains *N*-terminal phosphotyrosine-binding (PTB) domains and a *C*-terminal Rab-GTPase (GAP) domain. In response to insulin, TBC1D1 is phosphorylated by Akt and AMPK, however, this does not alter intrinsic Rab–GAP activity [72]. 

The efficacy of glucose uptake by cancer cells depends mainly on activities of GLUT1 and GLUT3 and, to a lesser extent, GLUT4 and GLUT10 [73]. For example, higher expression of GLUT1 and GLUT3 in papillary carcinoma, as compared to follicular carcinoma and non-neoplastic thyroid lesions, has been reported [74]. Additionally, both GLUT1 and GLUT3 have been up-regulated in poorly differentiated endometrial and breast cancers, both at mRNA and protein levels [75]. Transactivation of GLUT3 occurs in a Yes-associated protein (YAP)-dependent manner, suggesting that this pathway serves as a regulator of metabolic reprogramming during cancer progression, and thus can be considered as a promising anti-cancer therapeutic target [76].

GLUT1 localization in a cell membrane can be increased by phosphorylation of thioredoxin-interacting protein (TXNIP), an α-arrestin family protein. The elevated glucose concentration can induce expression of TXNIP, which inhibits glucose uptake directly by binding to GLUT1 and stimulating its endocytosis via clathrin-coated pits or indirectly by reducing GLUT1 biosynthesis at the mRNA level. Furthermore, AMPK has been shown to cause phosphorylation and rapid degradation of TXNIP, thereby increasing GLUT1 function [77]. In addition to gene expression regulation at the DNA level, histone modifications can contribute to modulation of glucose transporter induction. For example, epigenetic regulation of the expression of *SLC2A1* gene encoding GLUT1 can be due to the induction of *HDAC2* gene by beta-hydroxybutyrate, a ketone body, to enhance H3K9 acetylation under starvation conditions in brain tissue [78]. 

GLUT3 induction during epithelial-to-mesenchymal transition (EMT) by ZEB1 transcription factor to promote non-small cell lung cancer cell proliferation has been observed [79]. Additionally, in non-small cell lung carcinoma cell culture and in an in vivo model, increased glucose uptake with the involvement of GLUT3 and caveolin 1 (Cav1), an important component of lipid rafts, triggered tumor progression and metastasis. Interestingly, Cav1-GLUT3 signaling can be targeted by atorvastatin, an FDA-approved statin, which decreases cholesterol biosynthesis due to the inhibition of 3-hydroxy-3-methyl-glutaryl-CoA reductase, and this reduces EGFR-tyrosine kinase inhibitor (TKI)-resistant tumor growth and increases the overall patient survival [80].

The expression level of GLUT1 correlates with that of HIF-1α in many cancer types, including colorectal and ovarian cancers, and is associated with tumor clinicopathological characteristics such as tumor size, location, and patient age and gender; however, there can be differences in the intracellular location of these two proteins [81,82]. For example, GLUT1 was found in membranes of multifocally necrotizing cancer cells and in the cytoplasm of cancer cells with no necrosis, whereas HIF-1α mostly had a cytoplasmic location [82]. Immunoreactivity of GLUT1 was significantly higher in node-positive colorectal cancer compared to node-negative colorectal cancer. 

Additionally, an interplay between GLUTs, HIF-1, and glycolytic enzymes has been observed in many cancer types. For example, HIF-1α expression has been reported to correlate positively with those of both GLUT1 and LDH-5 at both mRNA and protein levels in human gastric and ovarian cancers, and this was found to be associated with tumor size, depth of invasion, distant metastasis, clinical stage, and differentiation status [83,84]. Additionally, correlation between the expressions of GLUT1, VEGF, and 6-phosphofructo-2-kinase/fructose-2,6-bisphosphatases-3 and -4 (PFKFB-3 and PFKFB-4) has been observed in gastric and pancreatic cancers. GLUT3 induction also correlates with the over-expression of glycolytic enzymes including HK2 and pyruvate kinase M2 (PKM2), which are associated with cancer invasiveness, metastasis, and poor prognosis [85].

## 4. Role of HIF-1 in Metabolic Reprogramming of Cancer Cells 

### 4.1. Enhancement of Glycolysis

As early as in 1925, C. Cori and G. Cori found glucose content was 23 mg less and content of lactate was 16 mg greater than those in veins of normal tissues when studying the axillary veins of hens with Rous sarcoma [86]. Afterwards, Otto Warburg and co-workers compared glucose and lactate concentrations in tumor veins and arteries and found 69 mg greater lactate in the vein blood than that in the same volume of aorta blood of rats with Jensen sarcoma, whereas glucose uptake by the tumor tissue was 52–70% and by normal tissues was 2–18% [9]. 

The Warburg effect has been experimentally confirmed by over-expression of glycolytic enzymes accompanied by deficit in OXPHOS-mediated ATP production in many cancer types in both cultured cell lines and animal models [87,88]. Genes affected by HIF-1 and implicated in carcinogenesis include *SCL2A* solute carrier family and those encoding glycolytic enzymes such as hexokinase II (HK II), phosphofructokinase 1 (PFK1), fructose-bisphosphate aldolase A (ALDOA), α-enolase (ENO1), pyruvate kinase M2 (PKM2), and lactate dehydrogenase A (LDH-A or LDH-5), as well as genes encoding PDK and enzymes of PPP [89,90]. 

**Figure 1:**
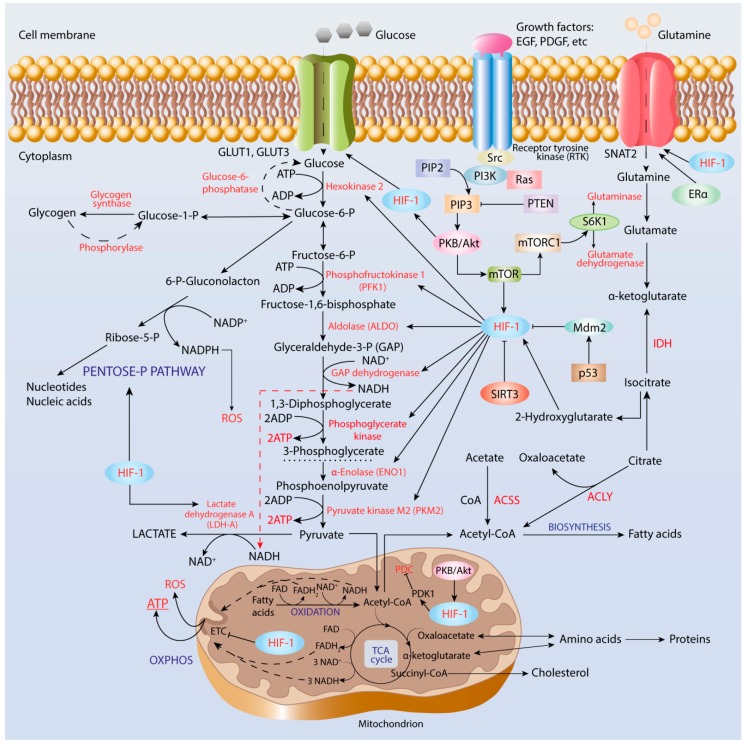
Regulation of metabolic reprogramming in cancer cells. Hypoxia-inducible factor-1 (HIF-1) induces expression of genes, which encode glucose transporters, GLUT1 and GLUT3, enzymes of glycolysis and pentose-phosphate pathway, and pyruvate dehydrogenase complex kinase. Activity of HIF-1 is regulated by Ras-protein kinase B (PKB)/Akt-mammalian target of rapamycin (mTOR) axis.

The first reaction of glycolysis (Figure 1) is catalyzed by the key rate-limiting enzyme, hexokinase, which has four isoforms in mammalian cells, among which HK II is over-expressed at both mRNA and protein levels in many tumor types including HCC, ovarian cancer, and others [91,92,93]. Furthermore, correlation of over-expression and co-localization of both HK II and HIF-1α in cancer cells near necrosis regions have been reported.

The second key rate-limiting glycolytic enzyme is PFK, a tetrameric enzyme in mammals that catalyzes the third reaction of glycolysis, that is, phosphorylation of fructose-6-phosphate to fructose-1,6-bisphosphate (FBP) accompanied by ATP utilization. Interplay between HIF-1α and Ras/Src oncogenes in tumor microenvironment in the regulation of PFK1 and PFK2 isoenzymes has been suggested as being a contributor to human cancer cell proliferation and survival [94]. PFK1 is an allosteric enzyme activated by fructose-2,6-bisphosphate that is produced from fructose-6-phosphate by the bifunctional phosphofructo-2-kinase/fructose-2,6-bisphosphatase (PFKFB) family of enzymes, which is induced by HIF-1α. PFKFB3 has the highest kinase activity, whereas PFKFB4 has more FBP-ase-2 activity and co-expression of PFKFB3 and PFKFB4 provides sufficient glucose metabolism. Thus, the PFKFB family enzymes are promising targets in anti-cancer therapy in order to combat tumor growth, invasion, and metastasis. For example, silencing the *PFKFB2* gene has been shown to significantly inhibit ovarian and breast cancer growth and to enhance paclitaxel sensitivity and patient survival [95]. 

In the glycolytic pathway, there are two enzymes that catalyze the transfer of phosphoryl group from a substrate to ADP, thereby producing ATP in a reaction of substrate-level phosphorylation, serving as an energy source in hypoxia conditions. The first enzyme is phosphoglycerate kinase (PGK), which catalyzes conversion of 1,3-diphosphoglycerate to 3-phosphoglycerate. Several single nucleotide polymorphism variants of PGK1 with decreased catalytic efficiency and thermodynamic stability due to the alterations in local protein conformation have been found in carcinoma cells [96]. The second enzyme is pyruvate kinase, which catalyzes the last reaction of glycolysis under aerobic conditions, that is, conversion of phosphoenol-pyruvate (PEP) into pyruvate, being allosterically regulated by fructose-2,6-bisphosphate. Four mammalian PK isoforms differing by regulation and tissue specificity, designated as PKM1, PKM2, PKR, and PKL, have been described [97]. Among them, PKM2 is expressed in embryonic, proliferating, and tumor cells, and has a role in progression of many cancer types such as ovarian, gastric, and lung cancers, among others. [98,99]. PKM2 up-regulation has been shown to occur through mTOR-mediated HIF-1α stabilization and c-Myc-heterogeneous nuclear ribonucleoprotein (hnRNP)-dependent regulation [100].

Glyceraldehyde-3-phosphate dehydrogenase (GAPDH) catalyzes the sixth reaction of glycolysis—oxidation of GAP to 1,3-biphosphoglycerate accompanied by reduction of NAD^+^ to NADH. Regulation of GAPDH expression in cancer cells is not obvious. For example, GAPDH regulation has not been found in Hep-1-6 mouse hepatoma, Hep-3-B and HepG2 human hepatocellular carcinoma, A-549 human adenocarcinoma, and HT-29 and HCT-116 colon cancer cell lines [101]. This indicates that GAPDH is not an attractive target in anti-cancer therapy and emphasizes the importance of proper choice of housekeeping genes for correct interpretation of experimental results. 

Indeed, analysis and interpretation of transcriptomic data with the use of Cytoscape network showed highly over-expressed glycolytic genes including HK2, PFKP, ENO2, SLC2A3, SLC16A1, and PDK1 in patients with clear cell renal carcinoma [102]. However, according to this study, some other glycolytic enzymes such as ALDOB, PKLR, PFKFB2, G6PC, PCK1, FBP1, and SUCLG1 were highly down-regulated. Therefore, a combination of several approaches including genomics, proteomics, metabolomics, and bioinformatics approaches should be used for the explanation of altered metabolic phenotype, bioenergetic signature, and increased glucose uptake resulting from both the activation of anaerobic glycolysis for cell proliferation and the impairment in mitochondrial functions of cancer cells [103]. 

### 4.2. Pentose Phosphate Pathway 

Other metabolic pathways can contribute to the Warburg effect by producing intermediates that fuel glycolysis. The over-expression of the PPP enzymes associated with HIF-1α stabilization and tumor progression has been reported as serving as an indicator of poor prognosis in cancer. Because PPP is linked to glycolysis, inhibition of PPP enzymes can serve as a promising strategy in anti-cancer therapy. For example, the ability of natural peptide carnosine to decrease the activities of PPP enzymes, as well as malate-aspartate and glycerol-3-phosphate shuttle mechanisms that carry electrons from glycolysis to ETC, has been observed in glioblastoma cell lines [104].

PPP is a process that takes place in the cytoplasm and contains two branches: (i) oxidative branch yielding ribose-5-phosphate used in nucleotide and nucleic acid biosynthesis, giving rise to NADPH utilized in fatty acid biosynthesis, and (ii) non-oxidative giving rise to glyceraldehyde-3-phosphate (GAP) and fructose-6-phosphate, both of which can enter glycolysis [31]. The first PPP reaction is oxidation of glucose-6-phosphate to 6-phosphoglucono-δ-lactone catalyzed by a key time-limiting NADP-dependent enzyme, glucose-6-phosphate dehydrogenase (G6PDH). Up-regulation of G6PDH has been found in many cancer types and has been considered as a promising target for anti-cancer therapy to overcome cancer cell chemotherapy resistance [105,106]. For example, in human clear renal cell carcinoma, an elevated glucose uptake and consumption, along with increased activity of G6PDH and concentration of PPP-derived metabolites including NADPH, have been observed [107]. 

The second oxidation reaction is conversion of phosphoglucono-δ-lactone into ribulose-5-phosphate catalyzed by 6-phosphoglucono-δ-lactone dehydrogenase (6PGD), which is also over-expressed in many cancer types including lung and ovarian cancers [108,109]. Up-regulated enzymes of non-oxidative branch of PPP, such as thiamine pyrophosphate (TPP)-dependent transketolase family enzymes TKTL, TKTL1 and TKTL2, have also been found in various cancer types, including breast, lung, gastric, endometrial, head, and neck cancer [110,111,112,113,114].

In addition to PPP, there are two NADP-dependent enzymes that produce NADPH: (i) IDH and (ii) decarboxylating malate dehydrogenase (malic enzyme). Both enzymes are associated with TCA cycle and tumor growth. Over-expression of both ME1 and ME2 isomers of malic enzyme has been found to cause reduction in tumor suppressor 53 level, however, down-regulation of ME2 caused more prominent increase in ROS generation and phosphorylation/activation of p53 by AMPK followed by senescence, as compared to ME1 [115]. Importantly, NADPH is an essential component of NADPH oxidases, which represent a major source of ROS and produce superoxide anion radical (O_2_•^−^) as a primary product [116].

### 4.3. Cancer Acidification and Its Role in Reverse to OXPHOS

Typically, cells grown under in vitro culture conditions experience oxygen concentrations of 20%, the condition denoted as normoxia [117]. However, under physiological conditions, oxygen concentration in peripheral tissues can vary from 3.0% to 7.4%, with an average value of about 5.0% (38 mm Hg), the condition denoted as physioxia [118]. Physiological oxygen concentration can transiently decrease under in vivo conditions due to various processes such as vasodilation and increase in blood flow [119]. Moreover, oxygen consumption rates can considerably vary between cell types depending on mitochondrial content and metabolic activity [117]. 

Under physioxia conditions, pyruvate produced during glycolysis undergoes oxidative decarboxylation by pyruvate dehydrogenase complex (PDC) to yield acetyl-CoA and NADH. Oxygen deficiency condition (oxygen concentration less than 2%) decreases the efficacy of mitochondrial ETC functioning to cause an increase in NADH/NAD^+^ ratio, which triggers conversion of pyruvate into lactate instead of its further oxidation by PDC. The over-production of lactate is due to activity of lactate dehydrogenase enzyme, which has five isoforms (LDH-1 to LDH-5) differentially expressed in normal tissues and up-regulated during carcinogenesis [120]. For example, increased glucose uptake, induction of glycolysis-related genes, excessive lactate production, and HIF-1α activation associated with aggressive phenotype and poor prognosis have been observed in patients with HCC and in Ewing sarcoma cells [121,122]. These observations have led to the conclusion that forcing cancer cells into mitochondrial oxidative metabolism can efficiently suppress tumor progression, whereas targeting glycolytic enzymes can be effective strategy to combat cancer growth. 

Tissue lactate accumulation, up to 30–40 mM, leads to acidosis (pH ≤ 6.8), which is a hallmark phenotypic feature of tumor microenvironment affecting tumor progression, invasion, and metastasis [123]. Normal cells cannot grow in an acidic microenvironment, however, acidosis is a necessary condition in order to promote cancer cell migration and invasion [124,125]. Both endogenous and exogenous lactate is essential for the activation of certain enzymes such as matrix metalloproteinases, as well as for regulation of the expression of oncogenes (Myc, Ras), transcription factors (HIF-1, E2F1), tumor suppressors (BRCA1, BRCA2), and cell cycle genes, as shown in MCF-7 breast cancer [126]. 

Tumor-derived lactate can inhibit activities of immune cells contributing to evasion of tumor cells of immune surveillance [127]. Elevated LDH-A level is a negative prognostic cancer biomarker and is implicated in the enhancement of immune suppressive cells such as myeloid-derived suppressor cells (MDSCs), tumor-associated macrophages (TAMs), and dendritic cells (DCs) [128]. However, lactate inhibits activities of cytotoxic T-lymphocytes (CTLs) and natural killer (NK) cells. For example, production of lactate by pancreatic cancer cells exerts immunosuppressive action due to inhibition of innate immune response via repressing cytotoxic activity of NK cells [129].

In addition to lactate, carbon dioxide produced in catabolic pathways such as PPP contributes to acidification of the tumor microenvironment. For example, in hypoxia conditions, tumor cells have been shown to produce more HIF-1-induced IX and XII isoforms of carbonic anhydrase, which catalyzes reversible hydration of carbon dioxide into bicarbonate and protons to contribute to intracellular acidification and tumor cell survival [130]. Moreover, in a mouse model of ductal carcinoma, in situ differences in levels of GLUT1 and carbonic anhydrase IX expression between normal and pre-cancer cells along with heterogeneity in intracellular pH values have been demonstrated [131].

However, a growing body of data evidence that tumor cells demonstrate increased proton export due to the up-regulation of proton transporters such as Na^+^/H^+^ exchanger 1 (NHE1), H^+^-lactate co-transporter, and monocarboxylate transporters (MCTs) to regulate intracellular pH values [132,133]. For example, MCT1 serves as a prominent pathway for lactate uptake by human cervical squamous cell carcinoma, mouse model of lung carcinoma, and xenotransplanted human colorectal adenocarcinoma cells [134]. Activities of these proton exchange systems represent additional adaptation and selection mechanisms, which enable emerging of chemoresistant cell clones and tumor progression and metastasis. Moreover, targeting these proton exchange molecules has potential in anti-tumor therapeutic strategies. 

Importantly, due to the activities of lactate shuttle mechanisms, lactate can serve as energy fuel, important gluconeogenic substrate, and signaling molecule [135]. For example, lactate can fuel TCA cycle, as shown in human non-small cell lung cancers [136]. In genetically engineered fasted mice lung and pancreatic cancer cells, the contribution of circulated lactate to production of TCA cycle intermediates exceeded that of glucose, however, glutamine contributed more greatly than lactate in pancreatic cancer [137].

Quantification of ATP amount produced via glycolysis and OXPHOS in nine randomly selected cancer cell lines demonstrated that in the lactic acidosis microenvironment (20 mM lactate, pH 6.7) ATP was generated almost twice as much by OXPHOS and almost four times less by glycolysis than that without lactic acidosis [138]. Moreover, glucose consumption was much greater in the lactic acidosis environment than that without lactic acidosis in the same tumor cell lines. Therefore, lactate accumulation can serve as a mechanism that underlies the reverse from glycolysis to OXPHOS to produce ATP in cancer cells.

### 4.4. Lipid Biosynthesis

Under hypoxic conditions, the up-regulation of enzymes involved in fatty acid and cholesterol biosynthesis including citrate synthase, fatty acid synthase (FASN), and 3-hydroxy-3-methylglutaryl-CoA reductase (HMGCR) is observed in many cancer types including pancreatic ductal adenocarcinoma (PDAC) and lung adenocarcinoma [139,140,141]. A common single carbon source for both fatty acid and cholesterol biosynthesis is acetyl residue activated and carried by co-enzyme A, acetyl-CoA, which is supplied under physioxia condition by PDC. However, under hypoxia condition, there are two different ATP-dependent reactions that produce acetyl-CoA to link glucose and fatty acid metabolism: (i) cleavage of citrate by ATP citrate lyase (ACLY) to give rise to oxaloacetate and acetyl-CoA, and (ii) ligation of acetate by co-enzyme A catalyzed by acetyl-CoA synthase (ACSS) [142,143]. 

ACLY is a key enzyme in cellular lipogenesis aberrantly expressed in many cancer types such as breast, liver, colon, lung, and prostate cancers. ACLY expression negatively correlates with tumor stage and differentiation, and this makes ACLY a potent target for anti-cancer therapy [144]. ACLY links cellular metabolism to histone acetylation and, thereby, plays an important role in epigenetic regulation of cell functions [145]. Under hypoxia conditions, histone acetylation and chromatin accessibility following acetate supplementation have been shown to promote cancer cell differentiation [146].

Acetate is a nutritional source of carbon used by cancer cells for fatty acid and phospholipid biosynthesis under hypoxic condition. A functional genomics study showed that the activity of ACSS2 contributes to cancer cell proliferation under lipid deprivation conditions [147]. ACSS2 expression is up-regulated during metabolic stress and correlates with cancer progression and metastasis. For example, in primary and metastatic brain tumors, only 50% of carbon can be provided by glucose, whereas acetate oxidation can occur simultaneously with glucose oxidation correlating with the expression of ACSS2 [148].

Additionally, cancer cell survival and metastasis can be maintained by lipid biosynthesis promoted by shift in glutamine metabolism from oxidation to reductive carboxylation. HIF-1 activation results in reduction of the activity of α-ketoglutarate dehydrogenase (α-KGDH) complex. α-KGDH catalyzes production of succinyl-CoA and NADH from α-ketoglutarate (α-KG) and NAD^+^, respectively, and is inhibited by ROS, succinyl-CoA, and an increase in NADH/NAD^+^ and ATP/ADP ratios [149]. α-KGDH is a key mitochondrial enzymatic complex in determining flux through TCA cycle; this leads to the activation of glutamine-dependent fatty acid biosynthesis [150].

Dietary lipids also can affect lipid metabolism and have a role in shaping the tumor micro-environment, as well as cancer progression and treatment [151,152]. High-fat diet-induced obesity and type 2 diabetes mellitus are considered as risk factors for many cancer types, including pancreatic and breast cancers [153,154]. High body mass index in individuals with no previously diagnosed tumor has been shown as being associated with high cancer risk [155]. In mice, high-fat diet stimulates oncogenic KRAS via cyclooxygenase 2 (COX2) activation, leading to pancreatic inflammation, fibrosis, and development of invasive PDAC [156], whereas fibroblast growth factor 21, a metabolic regulator preventing obesity, causes reduction in tumor growth [157].

## 5. Oxidative Metabolism and OXPHOS in Cancer

As Warburg wrote, “cancer cells can obtain approximately the same amount of energy from fermentation as from respiration, whereas the normal body cells obtain much more energy from respiration than from fermentation”, and uncoupling of respiration and phosphorylation with no diminishing oxygen consumption causes decrease in ATP production [10]. 

**Figure 2:**
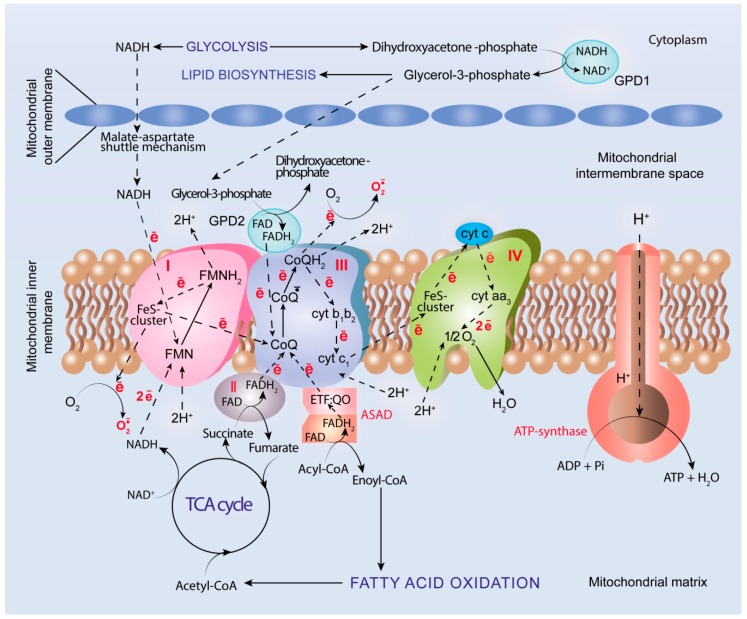
Oxidative metabolism, oxidative phosphorylation (OXPHOS), and reactive oxygen species (ROS) generation. NADH is mainly produced by glycolysis, pyruvate decarboxylase complex (PDC), fatty acid β-oxidation (FAO), and tricarboxylic acid (TCA) cycle to fuel electron transportation chain (ETC) via complex I, whereas FADH_2_ is mainly produced by FAO and TCA cycle and fuels ETC via complex III. Glycerol-phosphate and malate-aspartate shuttle mechanisms serve to transfer reducing equivalents through the outer mitochondrial membrane from the cytoplasm to ETC. Superoxide anion radical, a primary type of ROS, is produced as a byproduct of ETC.

Currently, it is obvious that molecular oxygen is an indispensable component of mitochondrial ETC and serves as a final acceptor of electrons transferred through ETC enzymatic complexes (I, II, III, and IV) localized in the inner mitochondrial membrane (Figure 2). The energy of electrons is used for ATP biosynthesis with the involvement of ATP-synthase (complex V) in the process denoted as OXPHOS [158]. A growing body of data evidence that the elevated oxidative metabolism with increased uptake of mitochondrial fuels such as lactate, pyruvate, and ketone bodies are characteristic for many cancer types including head and neck cancer, breast cancer, and lymphomas, among others [159,160,161]. For example, up-regulation of mitochondrial OXPHOS featured by succinate dehydrogenase (complex II) and cytochrome c oxidase (complex IV) activation, allowing them to produce a higher amount of ATP, has been observed in epithelial cancer cells [162,163]. Cancer stem cells resist glucose deprivation and over-express genes associated with oxidative metabolism including OXPHOS, PPP, and FAO, along with a higher level of ROS generation [164]. 

Additionally, chemotherapy has been shown to induce shift from glycolysis to OXPHOS mediated by SIRT1 and transcriptional co-activator PGC1 to promote tumor survival during treatment [165]. Several multidisciplinary approaches have been proposed to target mitochondrial functions and tumor cell metabolic plasticity as anti-cancer treatment strategies [166,167].

Two co-enzymes, NADH and FADH_2_, produced in reactions of oxidation of various biomolecules in the cytoplasm or in mitochondrial matrix, are the main suppliers of high-energy electrons for ETC. The major sources of NADH or FADH_2_ are FAO and oxidative degradation of glucose, which proceeds through the following three sequential metabolic processes: (i) aerobic glycolysis, which occurs in the cytoplasm through 10 enzymatic reactions to give rise to two molecules of pyruvate per one glucose molecule; (ii) oxidative decarboxylation of pyruvate by PDC to form acetyl-CoA, which further enters (iii) TCA cycle, which is a source of not only electrons, but also important intermediates such as α-ketoacids including α-KG and oxaloacetate utilized in biosynthesis of amino acids and other biomolecules [168]. 

### 5.1. PDC and TCA Cycle

Genetic and epigenetic alterations in PDC and TCA cycle enzymes promote metabolic shift in cancer cells from OXPHOS to glycolysis. Mutations in genes encoding aconitase, isocitrate dehydrogenase (IDH), succinate dehydrogenase (SDH), fumarate hydratase (FH), and citrate synthase have been observed in many cancer types [169]. Impairments in the expression of these enzymes result in accumulation of Krebs cycle intermediates named oncometabolites, which stabilize HIF-1 and nuclear factor-like 2 (Nrf2) transcription factors and ROS generation, while inhibiting p53, PDH3, and the PDC enzyme pyruvate dehydrogenase isoenzyme 3 (PDH3) [170].

Cancer-associated mutations in genes encoding IDH, FH, and SDH lead to the accumulation of 2-hydroxyglutarate, fumarate, and succinate, respectively [171,172,173]. Multiple mutations in IDH isoenzymes IDH1 and IDH2, which normally catalyze oxidative decarboxylation of isocitrate to α-KG, have been shown to occur frequently in gliomas and acute myeloid leukemia [174,175,176]. They lead to decrease in α-KG content and simultaneous increase in the amount of its antagonist, 2-hydroxyglutarate [177]. 2-Hydroxyglutarate, being accumulated in tumor cells, serves as a competitive inhibitor of multiple α-KG-dependent dioxygenases including histone demethylases and TET (ten-eleven translocation) family 5-methylcytosine (5mC) hydroxylases. Fumarate and succinate have also been proposed as acting as competitive inhibitors of α-KG-dependent oxygenases, including the HIFα hydroxylases contributing to HIF stabilization [178,179].

Nevertheless, cancer metabolome analysis has demonstrated that proliferating tumor cells require more diverse and large quantities of nutrients. Despite the earlier opinion that cancer cells bypass TCA cycle, emerging evidence increasingly demonstrates that many cancer cells rely heavily on this process in order to meet the requirements in nutrients and energy [180]. Moreover, most tumor cells can retain functional mitochondria and TCA cycle intermediates, which serve as substrates for nucleotides and nucleic acid, amino acid, and fatty acid biosynthesis [181].

Indeed, higher pyruvate uptake and mitochondrial activity associated with increased ATP production have been observed in more invasive ovarian cancer cells as compared to less invasive ones [182]. Additionally, activation of both PDC and TCA cycle enzymes with the production of about 50% acetyl-CoA from glucose, along with synthesis of glutamine and glycine from TCA cycle metabolites, have been observed in brain cancers both in humans and in animal models [183]. Furthermore, metabolic complexity, which includes oxidation of glucose to pyruvate followed by its oxidative decarboxylation to acetyl-CoA, which enters the TCA cycle along with glutamine oxidation, have been observed in a mouse in vivo model of genetically diverse primary human glioblastomas [184]. 

### 5.2. Glutaminolysis

Cancer cells demonstrate a higher rate of glutamine consumption as compared to normal cells, as glutamine catabolism can accommodate carbon and nitrogen demands for nucleotide and nucleic acid biosynthesis required for cell division and proliferation [185]. This takes place due to glutaminolysis, the catabolic pathway of glutamine degradation, in order to yield α-KG through the following reactions: (i) deamination of glutamine by glutaminase (GLS) giving rise to glutamic acid and ammonia, followed by (ii) oxidative deamination of glutamate by glutamate dehydrogenase (GDH), or (iii) glutamate transamination with alanine by alanine amino transferase and with aspartate by aspartate amino transferase. α-KG can further enter the TCA cycle, as it is an anaplerotic intermediate of this process and serves as energy fuel for cells [186]. 

Metabolic profiling studies showed that glycolysis is decoupled from TCA cycle in cancer cells, mainly through glutaminolysis to feed TCA as an alternative source of carbon (reviewed by [187]). Glycolysis yields lactate and pyruvate, the latter of which can be carboxylated by pyruvate carboxylase (PC) to oxaloacetate, and also anaplerotically fuels the TCA cycle for cancer growth and metastasis [188,189]. Thus, anaplerotic replenishment of TCA cycle in cancer cells depends on both glutamine degradation and glucose-derived pyruvate carboxylation to oxaloacetate. However, carbon can travel through TCA in the reverse direction to feed fatty acid biosynthesis, whereas lactate and pyruvate can be used in gluconeogenesis and for biosynthesis of non-essential amino acids [187]. Various anti-cancer therapeutic agents that improve glucose utilization are currently under pre-clinical and clinical testing. Among them, metformin is a potential anti-tumor drug that inhibits hepatic gluconeogenesis and can exert its effects via activation of AMPK [190]. Additionally, the metformin analogue phenformin, alone or in combination with gefitinib, inhibits bladder cancer growth via AMPK activation and EGFR signaling inhibition [191]. 

Under glutamine deprivation conditions, cancer cells have been shown to undergo oncogenic transcription factor c-Myc-driven up-regulation of GLS and GDH, as well as cell cycle arrest [192]. Moreover, activation of mTOR complex 1 and ribosomal protein S6 kinase-β1 (mTORC1/S6K1)-mediated pathway have been observed to regulate c-Myc to promote uptake of glutamine and to stimulate its catabolism via up-regulation of GLS in pancreatic cancer cells through modulating phosphorylation of eukaryotic translation initiation factor eIF4B, which is crucial to unwind its 5’-untranslated region (5’UTR) [193]. Activation of GDH proceeds through suppression of mitochondrial sirtuin SIRT4, which is over-expressed in human cancers [194].

Additionally, glutamine transporter SNAT2 (Figure 1) facilitates transfer of glutamine into cancer cells to promote their proliferation. In breast cancer cells, SNAT2 can be induced by both HIF-1α and estrogen receptor-α (ER-α), the binding sites for both HIF-1α and ER-α being overlapped in cis-regulatory elements of the SNAT2 gene [195]. Up-regulation of SNAT2 can cause complete resistance to anti-estrogen therapy and, partly, to anti-VEGF treatment, indicating that developing drugs targeting SNAT2 is a promising strategy in endocrine-resistance breast cancer. 

### 5.3. Fatty Acid β-Oxidation 

Triacylglycerols and fatty acids of adipose tissue are potential sources of feeding cancer growth. FAO is the most efficient metabolic pathway, producing NADH and FADH_2_ and proceeding in the mitochondrial matrix to yield acetyl-CoA, which further enters the TCA cycle, and this (i) provides a link between glucose and fatty acid metabolism, (ii) enables generation of a larger amount of ATP, and (iii) produces important intermediates used in other metabolic pathways [196]. FAO produces a greater amount of ATP per one substrate molecule through OXPHOS as compared to oxidative degradation of glucose. Up-regulation of FAO enzymes and their key roles in aerobic respiration have been observed in many cancer cell lines, including human malignant gliomas; HCC; and breast, lung, and ovarian cancers [197,198,199,200]. 

Indeed, metastatic triple-negative breast cancer (TNBC) cells maintain a high level of ATP production through the activation of mitochondrial FAO and carnitine-palmitoyltransferases 1 and 2 (CPT1 and CPT2), trans-membrane enzymes located in outer and inner mitochondrial membranes, respectively [201,202]. CPT1 is a rate-limiting enzyme that catalyzes interaction of acyl-CoA esters of long-chain fatty acids with carnitine to shuttle them into the mitochondrial matrix for β-oxidation [203]. It is allosterically inhibited by malonyl-CoA and exists in three isoforms: CPT1A (liver isoform), CPT1B (muscle isoform), and CPT1C (brain isoform) [204]. Over-expression of CPT1A correlates with cell cycle progression and the overall poor survival of ovarian cancer patients [201]. Oppositely, inactivation of CPT1A causes a decrease in ATP production and cell cycle arrest in G0/G1 phase due to activation of cyclin-dependent kinase inhibitor p21 by FoxO transcription factor phosphorylated/activated by AMPK and JNK/p38 MAPKs.

The inhibitory effect of malonyl-CoA is less efficient in relation to CPT1A as compared to CPT1B and, hence, active CPTA1 can present in cancer cells exhibiting a high rate of both FAO and fatty acid biosynthesis [205]. Indeed, in MCF-7 and MDA-MB-231 breast cancer cell lines, prolactin stimulates the over-expression of CPT1A at both mRNA and protein levels [206]. This is accompanied by increased phosphorylation of catalytic α-subunit of AMPK at Thr172 and acetyl-CoA-carboxylase (ACC) at Ser79, leading to enhancement of FAO to meet high energy requirements of cancer cells.

ACC catalyzes ATP-dependent carboxylation of acetyl-CoA to malonyl-CoA, a rate-limiting reaction in fatty acid biosynthesis, and ACC is expressed in two isoforms in mammalian cells—ACC1 and ACC2, differed by tissue distribution [207]. The enzymatic properties of ACC are complex, and its expression can be controlled at the level of transcription, which is sensitive to hormones and metabolic. Additionally, the activity of ACC can be regulated through multiple pathways, including phosporylation and low-molecular weight modulators [208]. AMPK- and liver kinase B1 (LKB1)-mediated phosphorylation leads to inactivation of ACC, whereas citrate, glutamate, and dicarboxylic acids can allosterically activate ACC. 

Our knowledge on molecular mechanisms underlying ACC functioning in cancer cells is limited. In HCC cells, hepatitis B virus X protein (HBx) expression under glucose deprivation conditions stimulates phosphorylation of AMPK and ACC to cause FAO activation, as well as increased ATP and NADH generation [198]. In subsets of cancer, including acute myeloid leukemia, down-regulation of PHD3 to enable greater utilization of fatty acids through FAO is observed. Conversely, the over-expression of PHD3 causes hydroxylation of ACC2 to suppress FAO [209].

Activation of FAO in TNBC cells is associated with activation of c-Src proto-oncogene tyrosine kinase via autophosphorylation at Y419 residue to promote tumor progression and metastasis [202]. Furthermore, a targeted metabolomic approach showed that significant up-regulation of FAO intermediates can occur in a c-Myc oncogenic transciption factor-dependent manner in TNBC cells [197]. Pharmacological inhibition of FAO dramatically decreases energy production in c-Myc-driven TNBC cells and blocks tumor growth. 

Lipid metabolism transition through suppression of lipogenesis and activation of FAO can associate with transforming growth factor β1 (TGF-β1)-induced EMT and cancer cell metastasis [210]. Over-expression of Snail1, a TGF-β1-induced EMT mediating transcription factor, inhibits fatty acid synthase and enhances mitochondrial respiration. Additionally, epigenetic-transcriptional regulation of fatty acid metabolism contributes to cancer growth and metastasis [211].

### 5.4. ROS Generation 

In addition to ATP production, mitochondrial ETC serves as a primary endogenous source of ROS (Figure 2) and generates superoxide anion radicals in large amounts, although they are generated as a byproduct rather than a primary product [212,213]. ROS generation by mitochondrial ETC results from leak of electrons and incomplete reduction of molecular oxygen to yield O_2_•^−^. In addition to well-recognized ETC sites of ROS generation, enzymatic complexes I and III, mitochondrial FAD-dependent glycerol-3-phosphate dehydrogenase (GPD2), and a system of electron transfer flavoprotein and electron transfer flavoprotein: ubiquinone oxidoreductase (ETF/ETF:QO system) have been identified. GPD2 is involved in the glycerolphosphate shuttle mechanism to carry reducing equivalents produced in glycolysis by cytoplasmic glyrerol-3-phosphate dehydrogenase (GPD1) through the outer mitochondrial membrane to ETC located in the inner mitochondrial membrane [214]. The ETF/ETF:QO system is involved in transferring of electrons from 11 different mitochondrial flavoprotein dehydrogenases, including FAD-dependent acyl-CoA dehydrogenases (ASADs), which catalyze dehydrogenation of acyl-CoA to enoyl-CoA during β-oxidation of fatty acids [215,216]. In ETC, both systems transfer electrons from FADH_2_ to CoQ to yield FAD and CoQH_2_, respectively, and to give one electron for incomplete reduction of O_2_ to O_2_•^−^. Thus, oxidative metabolism is associated with generation of ROS, which can both cause alterations in cellular redox homeostasis and underlie redox signaling in order to regulate cell response to stress stimuli. 

Various human cancer types produce much greater amount of ROS as compared to normal tissues (reviewed in [217]). Alterations in signal transduction pathways that control mitochondrial bioenergetics and dynamics cause mitochondrial dysfunction and elevated ROS production, which are implicated in determining cancer cell fate for survival or death [218]. Two mitochondrial phenotypes, ETC overload with preserved mitochondrial functions and partial ETC inhibition producing superoxide, have been observed in tumor cells. Both have c-Src and Pyk2 protein tyrosine kinases as downstream effectors, and switching between these mitochondrial phenotypes provides metastatic advantages for tumor cells [219].

Thus, ROS production contributes to tumor microenvironment, which is highly heterogeneous and can affect tumor growth by multiple means, depending on interplay between various intracellular and environmental factors, among which a key role belongs to AMPK.

## 6. Role of AMPK in Promoting Cancer Cell Oxidative Metabolism

AMPK is an energy and nutrient sensor activated in response to energy starvation to provide the restoration of ATP level in cells due to switch from anabolic to catabolic metabolism (reviewed by [220,221,222,223]. High AMPK activity is associated with variety of metabolic processes including stimulation of glucose uptake by cells and mitochondrial oxidative metabolism, that is, glucose oxidation, FAO, and OXPHOS. Additionally, AMPK activation leads to the inhibition of fatty acid and protein biosynthesis, cell cycle progression, and cell proliferation in both normal and tumor cells [221]. 

**Figure 3:**
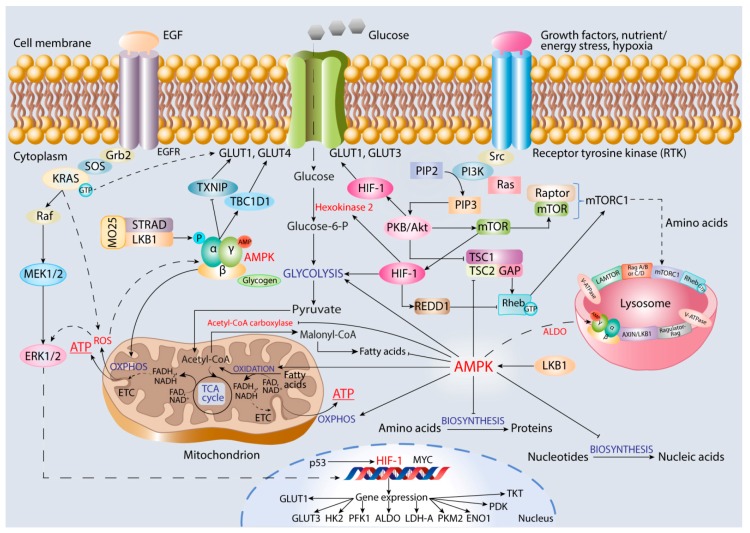
Interplay between AMPK-, HIF-1-, and ROS-regulated growth factor/nutrient and energy stress/hypoxia-initiated cell signaling pathways in controlling both glycolysis and OXPHOS to produce ATP for cancer cell proliferation, invasion, and migration. The involvement of AMPK in lysosomal complex formation is also shown.

AMPK is a heterotrimeric serine/threonine protein kinase expressed in different tissues and existing in various combinations of catalytic α-subunit and two regulatory β- and γ-subunits to provide diverse roles in regulating cell proliferation, autophagy, and metabolism [22]. At low ATP concentration, AMPK is allosterically activated by AMP/ADP binding in order to enable phosphorylation of specific enzymes. Adenine nucleotides bind to four tandemly arranged cystathionine-β-synthase (CBS) domains in the AMPK γ-subunit (Figure 3). Binding of AMP stimulates phosphorylation of Thr172 residue in the kinase domain of α-subunit by upstream kinases such as LKB1, which directly activates AMPK in response to energy stress [224]. LKB1 forms a complex with STRAD (STE20-related kinase adapter protein-α) and scaffolding protein MO25 in order to be activated [224,225]. The β-subunit contains glycogen-binding domain and allows AMPK accumulation in the large cytoplasmic inclusions.

In some cell types, AMPK activation can occur through the AMP/ADP-independent mechanism, which does not require an intact AMP-binding site. For example, fructose-bisphosphate aldolase (ALDO), a sensor of glucose availability, when occupied by its substrate, fructose-1,6-bisphosphate, cannot promote AMPK activation, whereas ALDO free of FBP stimulates the formation of a lysosomal complex composed of AMPK, V-ATPase, Ragulator, AXIN, and LKB1 kinase tumor suppressors; this complex is required for AMPK phosphorylation and activation by LKB1 [226]. An inhibition of LKB1–AMPK signaling by G6PD activation and ribulose-5-phosphate formation in PPP followed by activation of ACC to provide a link between PPP, lipid biosynthesis, and tumor growth has been observed [227].

With the use of comprehensive proteomics, phospho-proteomics, and systems biology approaches, a large network of AMPK activator and substrate proteins involved in cell migration, adhesion, and invasion has been identified [228,229,230]. One of the key downstream signaling pathways regulated by AMPK is mTOR-mediated signaling, which controls cellular response to environmental stress stimuli through the formation of two distinct complexes, mTORC1 and mTORC2 [231]. mTORC1 is sensitive to changes in cell growth conditions and contains scaffolding Raptor protein and mTOR to trigger anabolic metabolism, that is, protein, lipid, and nucleic acid biosynthesis (Figure 3). mTOR can be regulated by growth factors, as well as changes in cellular energy and nutrient concentrations, to control numerous cellular processes at both transcriptional and translational levels. Importantly, mTOR-mediated signaling is integrated with PKB/Akt, HIF-1, and AMPK signaling pathways to control cell proliferation and survival in nutrient and energy deprivation conditions [232]. 

AMPK can inhibit mTORC1 through direct phosphorylation of several residues, including Ser1387 in tumor suppressor TSC2, which forms heterodimeric complex with TSC1 for the activation [233]. TSC1–TSC2 complex relays signals from diverse cellular pathways to properly modulate mTORC1 activity [234,235]. TSC2 contains GTPase-activating protein (GAP) domain, which activates small GTPase Ras homolog enriched in brain (Rheb), which in turn in its active, GTP-bound form can activate mTORC1. Rheb and Rag small GTPases act together to localize mTORC1 to lysosomal membrane and Ragulator complex in response to amino acids to activate mTORC1 and to drive maturation of endosomes into lysosomes [236]. Rag GTPases form A/C and B/D heterodimers, which use a unique mechanism to stabilize their active (^GTP^RagA-Ragc^GDP^) or inactive (^GDP^RagA-Ragc^GTP^) states. Ragulator and lysosomal protein SLC38A9, an arginine sensor, are guanine exchange factors (GEFs) that control nucleotide loading state [237].

The Warburg effect can be closely associated with interplay between HIF-1 stabilization and decrease in AMPK activity, which underlies cancer cell survival and chemoresistance. In tamoxifen-resistant LCC2 and LCC9 breast cancer cell lines, rate of glycolysis is higher than that in MCF-7S cells, and HIF-1 was found to be activated through the Akt/mTOR signaling pathway, with phosphorylated AMPK being decreased without hypoxic conditions. The specific inhibition of glycolytic enzyme HK2 is associated with suppression of the Akt/mTOR/HIF-1 axis, and this, along with increase in AMPK activity, led to reduced lactate accumulation and cell survival [238].

Using a combination of mathematical modeling, systems biology, bioinformatics, and experimental data, an association between ROS, HIF-1, and AMPK activities in breast cancer cell lines has been shown. Systems biology approach with the use of AMPK/HIF-1/ROS circuit allowed for the prediction of three stable metabolic phenotypes of cancer cells: (i) glycolysis phenotype with high HIF-1 and low AMPK activities, (ii) OXPHOS phenotypes with high AMPK and low HIF-1 activities, and (iii) hybrid glycolysis/OXPHOS phenotype with both high HIF-1 and AMPK activities [239]. Hybrid metabolism can be maintained by elevated mitochondrial and NOX-mediated ROS production and regulation of c-Myc and c-Src oncogenes.

The hybrid metabolic phenotype enables more flexibility in utilizing a variety of nutrients in order to adapt to a very heterogeneous tumor microenvironment [240]. Improved analysis of the three stable steady states includes phosphorylated AMPK (pAMPK): HIF-1^high^/pAMPK^low^, HIF-1^low^/pAMPK^high^, and HIF-1^high^/pAMPK^high^, whereas OXPHOS phenotype includes oxidative glucose degradation and fatty acid β-oxidation. Analysis of well-annotated metabolomics and transcriptomics data along with mRNA sequencing data reported an association between HIF-1/AMPK activities and aggressive metastatic phenotype [23]. The authors concluded that targeting both glycolysis and OXPHOS is necessary in order to combat cancer aggressiveness.

Over-expression of AMPK contributes to tumor progression through multiple means, including stimulation of EMT and cell migration and adhesion. Moreover, AMPK signaling can exert opposite effects on tumor growth, depending on cancer cell microenvironment, including ROS production (reviewed in [241]). Therefore, the ability of AMPK to regulate cellular metabolism and to inhibit cancer growth can involve both nuclear- and mitochondria-mediated processes [242]. 

AMPK is a major controller of fatty acid metabolism. It inhibits acetyl-CoA carboxylase by phosphorylation ACC1 at Ser79 and ACC2 at Ser212 [243]. Additionally, malonyl-CoA inhibits CPT1 found in mitochondrial membrane to facilitate fatty acid entry into mitochondria associated with increased FAO [244]. On the other hand, elevated production of ROS and inhibition of AMPK by isorhamnetin, which triggers cell cycle arrest at G2/M phase due to increase in the expression of cyclin-dependent kinase (Cdk) inhibitor p21^WAF1/CIP1^, have been observed. Additionally, isorhamnetin-induced apoptosis is associated with down-regulation of Fas/Fas ligand, reduced ratio of B-cell lymphoma 2 (Bcl-2)/Bcl-2 associated X protein (Bax) expression, release of cytochrome *c* from mitochondria, and activation of caspases [245]. In human cholangiocarcinoma, up-regulation of uncoupling protein 2 (UCP2) associated with increased glycolysis, lymph node invasion, and poor prognosis have been observed [203]. In UCP2 down-regulated cells, AMPK is activated and the increased mitochondrial ROS generation and AMP/ATP ratio can be held responsible for this activation [246]. 

Additionally, gemcitabine has been shown to induce ROS/KRAS/AMPK-mediated metabolic reprogramming, mitochondrial oxidation, and aerobic glycolysis in order to promote stem-like properties of pancreatic cancer cells [247]. The subpopulation of dormant tumor cells with mutant KRAS oncogene has features of cancer stem cells and relies on mitochondrial respiration and OXPHOS, demonstrating decreased dependence on glycolysis as a source of energy [248,249]. Small GTPse KRAS is involved in Ras-MAPK-mediated signal transduction and formation of its active, GTP-bound form is dramatically increased by GAP. It activates c-Raf and can contribute to the Warburg effect in cancer cells through up-regulation of GLUT1. Enhanced glucose uptake and glycolysis rate along with increased cell survival associated with GLUT1 up-regulation has been observed in colorectal cancer cell lines with mutations in *KRAS* and *BRAF* genes under glucose deprivation conditions [250].

Smolkova and co-authors hypothesized that there may be waves in metabolic changes during carcinogenesis, which start from alterations in oncogene expression, and are followed by HIF-1 stabilization and metabolic reprogramming characterized by increased glycolysis and suppression of mitochondrial oxidation and OXPHOS [251]. High rate of cell proliferation causes hypoxia, as well as nutrient and energy deficiency, and this stimulates oxidative glutaminolysis and the involvement of LKB1-AMPK-p53 and PI3K/Akt-mTOR signaling, along with c-Myc dysregulation [252]. This leads to resumption of mitochondrial OXPHOS, and each type of neoplasm is characterized by distinct metabolic phenotype according to waves of metabolic changes and oncogenic mutations.

## 7. Conclusions 

Cancer is a complex disorder that is dependent on multiple intracellular, micro-environmental, and external factors for growth, invasion, and metastasis. The cancer microenvironment is highly heterogeneous and is characterized by both hypoxia and physioxia. This requires the involvement of numerous regulatory proteins to control tumor cell proliferation, differentiation, and migration. Under hypoxia conditions, HIF-1α serves as a key oxygen sensor and a major transcriptional regulator of numerous genes involved in glucose uptake and metabolism in order to provide switch in ATP production from OXPHOS to glycolysis. However, in many cancers, a reverse from glycolysis to oxidative mitochondrial metabolism has been observed. This requires an interplay of HIF-1α with another master regulator of ATP production, AMPK, which enables a switch from anabolic to catabolic metabolism via triggering oxidative degradation of glucose and β-oxidation of fatty acids, the major producers of NADH and FADH_2_ as sources of electrons for OXPHOS. This interplay involves growth factor-initiated signaling pathways, oncogenes, and transcription factors, and these multiple cross-talks underlie uncontrolled cancer growth, invasion, and metastasis, as well as cancer chemoresistence to conventional anti-tumor drugs. Comprehensive genomics, proteomics, bioinformatics, and systems biology approaches enable an understanding of cancer complexity and numerous interactions between various signaling pathways. However, more investigations are needed to elucidate mechanisms underlying the switch between oxidative and reprogrammed metabolism and cancer cell tolerance to micro-environmental changes for proliferation and migration. Multiple metabolic switches should be also taken into account in the discovery of novel molecular targets for anti-cancer agents. 

## Abbreviations

HIF-1	hypoxia-inducible factor-1
AMPK	AMP-activated protein kinase
GLUT	glucose transporter
ETC	electron transportation chain
OXPHOS	oxidative phosphorylation
PDC	pyruvate dehydrogenase complex
PDK	pyruvate dehydrogenase complex kinase
PFK	phosphofructokinase
PK	pyruvate kinase
LDH	lactate dehydrogenase
TCA	tricarboxylic acid
EGF	epidermal growth factor
VEGF	vascular endothelial growth factor
PDGF	platelet-derived growth factor
PKB	protein kinase B
mTOR	mechanistic (mammalian) target of rapamycin
UPS	ubiquitin-proteasome system
